# Next-generation tools to control biting midge populations and reduce pathogen transmission

**DOI:** 10.1186/s13071-020-04524-1

**Published:** 2021-01-07

**Authors:** Phillip Shults, Lee W. Cohnstaedt, Zach N. Adelman, Corey Brelsfoard

**Affiliations:** 1 Texas A&M University, 370 Olsen Blvd, College Station, TX 77843 USA; 2 Texas Tech University, 2901 Main St, Lubbock, TX 79409 USA; 3 USDA-ARS Arthropod Borne Animal Disease Research Unit, 1515 College Ave, Manhattan, KS 66502 USA

**Keywords:** *Culicoides sonorensis*, *Wolbachia*, IIT, SIT, Population suppression, Population replacement, Pathogen transmission

## Abstract

Biting midges of the genus *Culicoides* transmit disease-causing agents resulting in a significant economic impact on livestock industries in many parts of the world. Localized control efforts, such as removal of larval habitat or pesticide application, can be logistically difficult, expensive and ineffective if not instituted and maintained properly. With these limitations, a population-level approach to the management of *Culicoides* midges should be investigated as a means to replace or supplement existing control strategies. Next-generation control methods such as *Wolbachia*- and genetic-based population suppression and replacement are being investigated in several vector species. Here we assess the feasibility and applicability of these approaches for use against biting midges. We also discuss the technical and logistical hurdles needing to be addressed for each method to be successful, as well as emphasize the importance of addressing community engagement and involving stakeholders in the investigation and development of these approaches.

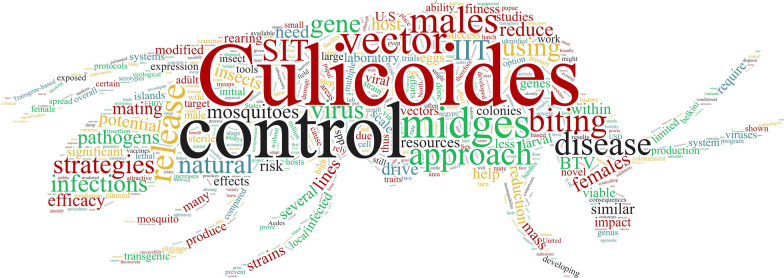

## Background

Biting midges in the genus *Culicoides* are small hematophagous insects that feed on a variety of vertebrate hosts. *Culicoides* midges are responsible for transmitting over 110 viral, protozoan and filarial pathogens worldwide [1, 2]. The diseases caused by these pathogens are of veterinary, medical and ecological importance, and include bluetongue (BT), epizootic hemorrhagic disease (EHD), African horse sickness virus (AHSV), Schmallenberg disease, and Oropouche fever [3, 4]. Multiple outbreaks of bluetongue virus (BTV) of different serotypes, topotypes (regional variants of particular serotypes) and strains have been recorded in Europe in recent decades [5, 6]. One of the largest European outbreaks to date resulted in economic damage greater than US$150 million (USD) in the Netherlands alone [7]. While severe disease outbreaks can cause a substantial loss in livestock numbers, their main economic impact stems from international trade restrictions and bans [8]. Worldwide estimates of direct and indirect losses due to just BT have been estimated to top USD 3 billion annually [9].

Methods of treatment and prevention for *Culicoides-*transmitted pathogens are broad and untargeted, or reactive to an outbreak, resulting in insufficient population reduction to prevent transmission [10, 11]. Current management practices for biting midges use a combination of broad-spectrum pesticide applications, larval habitat source reduction and behavioral management of livestock [12, 13]. Implementing these strategies over a large area can be difficult, expensive and/or harmful to the environment. The availability of vaccines is also limited for many diseases caused by *Culicoides*-transmitted pathogens. Attenuated vaccines are available for BTV, although their effectiveness varies as they often only protect against a single serotype [3, 12]. Inactivated viral vaccines for BTV have also been shown to be effective, but would be expensive for large-scale livestock applications in enzootic areas [14, 15]. With the concerns and limitations of the current control methods, research efforts are sorely needed to develop environmentally friendly and sustainable methods for *Culicoides* midge control.

The use of autocidal and next-generation control methods is an attractive option for implementation in *Culicoides* systems to reduce or replace natural populations and prevent disease transmission. These population-level control techniques utilize the biology of the target species to reduce the total number of vectors in a population [16, 17]. Suppression methods inhibit a target organism’s ability to produce viable offspring through the release of sterile or incompatible males. This reduction in potential vectors is presumed to lead to a reduction in pathogen transmission. Conversely, methods used for population replacement aim to lower virus transmission by reducing the vector competency of individuals within the population. Replacement strategies have garnered significant attention for the control of dengue virus (DENV) transmission in the *Aedes aegypti* mosquito [18–21]. Individuals resistant to pathogen transmission can be released into the environment until the disease refractory phenotype reaches fixation, thus replacing the wild population with one that has a limited ability to transmit pathogens.

*Culicoides sonorensis*, an important vector of BT and EHD viruses in North America, is a well-studied species with a significant number of molecular resources available, making it a prime candidate for the investigation of next-generation control methods [22]. *Culicoides sonorensis* has been reliably maintained in colonies for over 60 years, and existing rearing protocols can be scaled for mass production and releases [23, 24]. The genome of *C. sonorensis* has been published along with several reference transcriptomic studies [25–28], and further annotation and chromosomal mapping/assembly will help to maximize the utility of these resources. There are also several cell lines of *C. sonorensis* which will aid in the screening process for effector genes or *Wolbachia* strains that might interfere with pathogen replication prior to *in vivo* experiments [22]. Here we report our assessment of the potential application of autocidal, genetically and *Wolbachia*-based control techniques to reduce biting midge populations and as methods to limit pathogen transmission using *C. sonorensis* as a model. The outcome of initial tests within this more tractable species will help inform whether significant resources should be allocated to developing similar control methods in other *Culicoides* vector species.

## Management tools

### Sterile insect technique

**Fig. 1.**
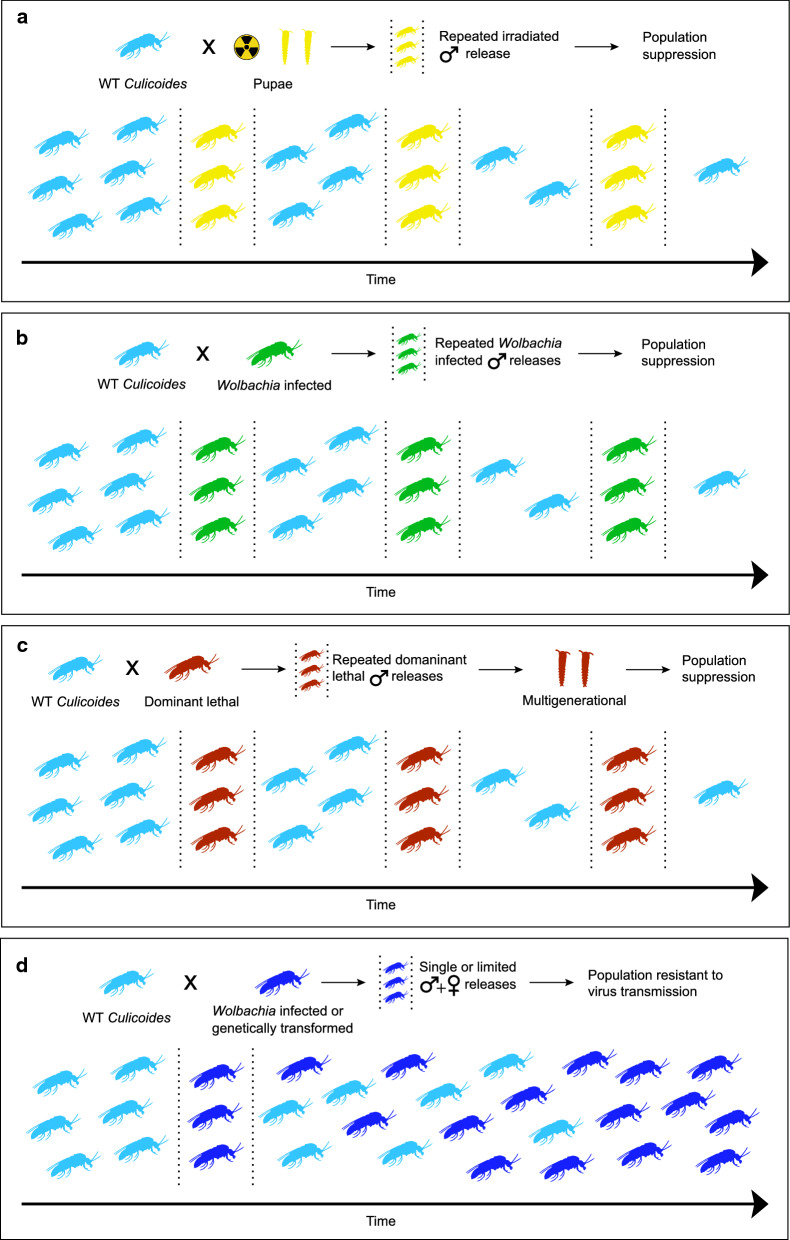
Proposed suppression and replacement approaches for *Culicoides* population and disease control. **a** Sterile insect technique (SIT) approach, **b** *Wolbachia*-based incompatibly insect technique (IIT) approach, **c** male-dominant lethal population suppression and **d** *Wolbachia-*based population replacement and genetic modification gene drive approaches. In all figures, the light-blue *Culicoides* midge symbols represent wild-type (*WT*) individuals

The sterile insect technique (SIT) is an autocidal, or “self-killing”, approach to pest control based on the mass inundative releases of irradiated sterile males. When irradiated males mate with wild females, the lack of viable sperm transferred ultimately causes the reduction of natural populations, provided the releases are sustained [29, 30] (Fig. 1a). SIT approaches have been used successfully to control *Cochliomyia hominivorax* (primary screwworm), *Glossina austeni* (tsetse fly) and *Ceratitis capitata* (medfly) [31–33], and are an attractive option for vector control as these released males do not negatively impact the host via blood-feeding or by transmitting pathogens. Additionally, this approach is environmentally friendly as it is species-specific and self-limiting [34]. As an initial step towards the development of an SIT approach targeting biting midges, Jones [23] exposed males and females from the USDA “AA” colony line of *C. sonorensis* to varying amounts of gamma radiation. Sterility of 95–100% was observed in males exposed to 10,000–15,000 rad, and this infertility lasted for up to five subsequent matings. It should be noted that Jones lists his measure of radiation dosage as “R” which could be either rads or roentgens. Females exposed to these doses showed a drastic decrease in the number of eggs laid. Jones [23] also demonstrated sterilization of pupae, although in many cases a higher dose (20,000–30,000 rad) was needed to prevent males from recovering fertility. A potential advantage to irradiating pupae is that fewer adverse side effects might be associated with transporting pupae as compared to the more fragile adult stages, similar to reports from shipping adult mosquitoes [35]. Even though *C. sonorensis* were exposed to relatively high amounts of radiation, little to no somatic damage was observed [23, 24]. Further studies are needed to fully investigate the use of SIT to control *Culicoides* midges; however, its simplicity and success in controlling other Dipterans makes this an promising approach.

### *Wolbachia*-based strategies

*Wolbachia* is an obligate intercellular bacterium found in a multitude of insect orders that is estimated to infect up to 55% of insect species [36]. *Wolbachia* has been demonstrated to cause reproductive phenotypes in its infected hosts, including male-killing, feminization of genetic males, parthenogenesis and cytoplasmic incompatibility (CI) [36]. The most well-studied reproductive modification is CI because of its applicability for insect vector control. CI results when a male infected with *Wolbachia* mates with an uninfected female or a female with a different *Wolbachia* infection type. The result of CI is that females in incompatible crosses produce non-viable offspring (i.e. eggs that do not hatch). Low-density *Wolbachia* infections naturally occur in wild populations of several species of *Culicoides* midges in Europe, Australia and the USA [37–39]. *Wolbachia* infections have also been demonstrated in several mosquito species to induce disease refractory phenotypes [40–44]. If *Wolbachia* strains that induce CI or pathogen refraction in their *Culicoides* hosts can be identified and transfected into important vectors, *Wolbachia*-based strategies may be a viable approach for use against *Culicoides* midges.

#### *Wolbachia*-based IIT

*Wolbachia-*based IIT approaches are based upon mass releases of incompatible *Wolbachia*-infected males, which can lead to suppression and potential elimination of a localized vector population (Fig. 1b) [45]. Similar to SIT, IIT also shares the same limitations of relying on consistent mass rearing and release of only males, although there is no need for specialized irradiators or radioactive materials for sterilization since *Wolbachia* induces CI. Fluorescent *in situ* hybridization experiments have shown localization of *Wolbachia* infections in the midgut, testes and ovaries of *C. sonorensis* [39]. The localization of *Wolbachia* infections in the reproductive tracts of *C. sonorensis* is suggestive that *Wolbachia* may be influencing the reproductive system of its *Culicoides* host [46]. Furthermore, infections identified in *C. sonorensis* are in similar *Wolbachia* clades that result in CI in other insects [46]; however, *Wolbachia*-induced CI or other reproductive phenotypes remain undocumented in any *Culicoides* spp. Additional studies are needed to examine for *Wolbachia*-induced CI among *Culicoides* species harboring natural *Wolbachia* infections. Field and laboratory trials have shown promising results by reducing mosquito populations in several species [47, 48]. Resulting technology and lessons learned from these studies can be used to help adapt IIT for use against *Culicoides* midges.

#### *Wolbachia*-based population replacement

Particular *Wolbachia* variants (e.g. the *w*Mel strain) partially block DENV, chikungunya virus, Zika virus and yellow fever virus transmission without impacting *Aedes aegypti* fitness [40–44]*.* As *Wolbachia*-infected females can mate and produce viable offspring with infected and uninfected males alike, their resultant reproductive advantage can drive a given disease refractory phenotype into a natural population (Fig. 1d). Releases of *Wolbachia*-infected mosquitoes by the World Mosquito Programs are ongoing in 15 countries, with a focus on reducing DENV transmission (www.eliminate.dengue.com). These releases have been remarkably successful at replacing natural populations with *Wolbachia*-infected individuals and are showing reductions in DENV transmission [49]. It is presumed that these W*olbachia* infections are directly competing with the pathogens for intracellular resources or the infection is resulting in an upregulation of the host’s immune system. Either or both of these could, in turn, influence the pathogen in the insect host [45, 50]. Recent transfection of *C. sonorensis* cell lines with a novel *Wolbachia* type suggest an upregulation of the host immune system, which may be associated with a pathogen-blocking phenotype; however, this needs to be tested *in vivo* [51]. Population replacement approaches are an attractive option for *Culicoides* disease control as they do not require the continued release of individuals after the desired phenotype reaches fixation in a population, although this self-sustainment also increases ecological concerns. After the establishment of *Wolbachia*, restoring the natural population or eliminating the introduced population may be difficult in the event of any undesirable outcomes [52].

### Transgene-based strategies

Whereas SIT elicits sterility via chromosomal damage to the reproductive cells, and certain *Wolbachia* species cause CI, infertility can also be induced molecularly through genetic modifications. Manipulating an insect’s genome through the insertion of genes or altering the expression levels of existing genes can produce individuals with a desired genotype [53, 54]. Local vector populations can be suppressed or pathogen transmission can be blocked by releasing genetically modified (GM) individuals carrying a lethal or pathogen-resistant transgene (reviewed in [55]). Autocidal or *Wolbachia*-based approaches rely on the disruption of fertilization or early embryonic development, whereas transgene-based approaches allow more control over the timing of gene expression and any associated consequences. For example, a lethal transgene can be designed to activate only during the pupal stage. This means the released GM individuals can develop normally as a larva and actively compete with wild types for resources, potentially increasing the power of the approach. Genetic engineering can also be used in conjunction with conventional SIT or IIT [56, 57], although the designation of the organisms as GM will affect when and where this strategy can be used. Transgene-based control methods will face some of the same logistical challenges associated with mass rearing and release, but they will also face public resistance due to the designation of these insects as genetically-modified organisms (GMO). In fact, the use of GMOs are banned outright in some countries, a situation unlikely to change in the foreseeable future.

Currently, methods for the genetic modification of *Culicoides* midges have not yet been described; however, tools such as CRISPR (clustered regularly interspaced short palindromic repeats)-Cas9 [58] and the broadly active transposable element *piggyBac* [59] are likely to be effective in biting midges, given their success in many other Diptera. Once validated methods are in place, genetic modifications proposed as a means to control other vector populations, such as mosquitoes, can be used as a template to create similar strains of transgenic *Culicoides* midges [60, 61]. Additionally, without a phylogeny for the genus, it will be hard to predict what information will be transferable between or with subgenera. As gene families can evolve independently between divergent groups, the genes associated with pathogen transmission or midge reproduction/development may be highly variable within the genus. Separate transgenic suppression or replacement methods may need to be developed for specific midge species, further complicating this approach.

#### Self-limiting transgene-based population suppression

Genetic control techniques such as RIDL (Release of Insects carrying a Dominant Lethal) can be adapted for use in *Culicoides* [55] (Fig. 1c). Males carrying a dominant lethal transgene are mass-released into a population with all of their progeny inheriting a copy of this transgene. The female offspring will subsequently die; however, the male offspring will survive and pass this transgene to 50% of their progeny offering multigenerational control. To maintain transgenic lines within the production facility, this approach requires the development of an inducible sex-lethal system to turn off expression of the lethal gene, similar to the Tet-on/off [62]. These lines can also be integrated into an SIT or IIT approach as a means of improving the speed and accuracy of sex-separation.

#### Transgene-based population replacement and gene drive

Certain *Wolbachia* spp. are capable of overcoming normal Mendelian inheritance, with the result that these strains can increase in frequency in the host population without actually offering a benefit or selective advantage. Similar methods of increasing transgene frequency in wild populations have been proposed to spread engineered transgenes to be used in population replacement approaches; these are termed gene drive (reviewed in [63]). A wide array of gene drive architectures have been developed in other Diptera, such as *Drosophila* [19, 64, 65] and mosquitoes [66–69]. These architectures will be helpful for the development of any future *Culicoides* midge population replacement strategy as the general principles of pathogen resistance and gene drive will be the same [50, 70, 71] (Fig. 1d). The effects of natural *Wolbachia* infections in target *Culicoides* species on a gene drive approach could also be nullified by the development aposymbiotic strains via antibiotic treatments. Any population replacement strategies developed using a gene drive system can be expected to vary in terms of persistence and invasiveness in the environment, and thus proper risk assessment and community engagement will be vital before any field-based evaluation or implementation [45, 72].

## Research gaps concerning the use of novel control approaches against *Culicoides*

### Investigation of sterility, CI, genetic modification and pathogen-blocking phenotypes

**Fig. 2.**
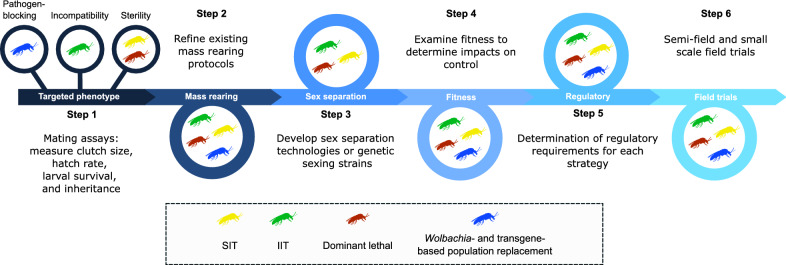
Systematic steps for future research to address hurdles for the development of IIT, population replacement, genetically-based and/or SIT approaches

The next-generation management techniques mentioned above rely on the creation of a targeted phenotype that is subsequently released into natural populations. The mechanisms underlying these approaches all vary; however, each will follow similar steps during development (Fig. 2). Solutions for overcoming hurdles in one strategy will likely translate to the others. Mating assays are often used to test the efficacy of a created phenotype, measuring clutch size, hatch rate, immature survival and inheritance [45, 67]. Such assays can only be performed if the target species mates under laboratory conditions, and many *Culicoides* spp. form mating swarms at established landmarks (environmental structures, larval habitats, the host, etc.) [73]. As colonies of *C. sonorensis* exist, laboratory mating assays should not impede the development of next-generation control strategies against this species.

**Fig. 3.**
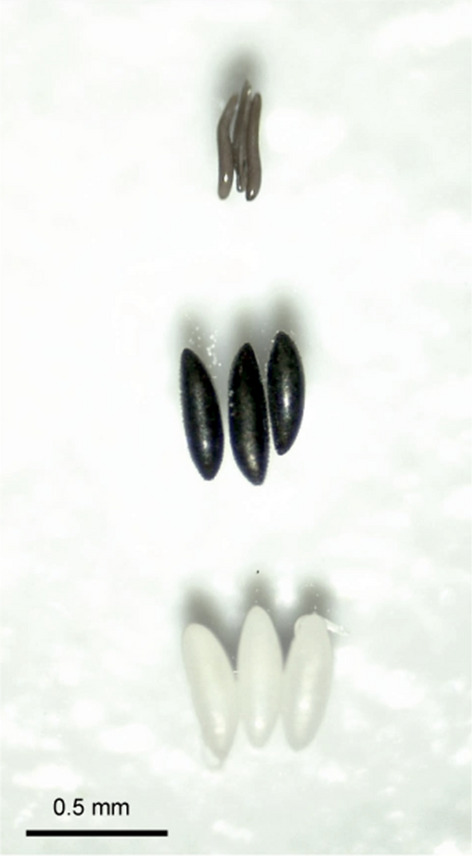
Side by side comparison of *Culicoides* eggs compared to eggs of other Diptera. Species eggs are displayed from top to bottom: *Culicoides sonorensis*, *Aedes albopictus* and *Drosophila melanogaster*, respectively. Magnification: ×40

Further work is needed to refine the radiation sterilization of *C. sonorensis* using modern methods and equipment, but such work can be started immediately. To introduce a *Wolbachia* strain or perform genetic modifications, a protocol for the microinjection of biting midge eggs must be established. While there are well-established protocols for microinjecting mosquito eggs [74] and *Drosophila* and sandfly eggs [75], *Culicoides* eggs are more elongated and have less volume, and are thus anticipated to be more difficult to manipulate (Fig. 3). Until successful microinjections can be conducted, this will be a barrier to the implementation all *Wolbachia-* and genetically-based strategy.

In developing new protocols for genetically-modified biting midges, we anticipate that the initial modifications would be the insertion of a marker gene, such as green fluorescent protein (for transposon-based approaches), or easily scorable visible markers, such as white-eyes (for CRISPR/Cas9 based approaches) [76, 77]. Following the validation of injection methods and transposable element (TE) integrations, the TE-based random insertion of candidate transgenes will help determine optimal integration site [78]. Such sites could be re-used using targeted recombinases [79] or CRISPR-Cas9 [80]. Research suggests that BTV vector competence is associated with the expression of glutathione S transferase (GST) and the antiviral helicase (SKi2) [28, 81]; thus, altering the expression of the two genes encoding these proteins with inserted promotors or suppressors may be a first step towards a genetically-based control strategy.

Many different cell lines derived from *C. sonorensis* exist, and their susceptibility to a number of BTV and epizootic hemorrhagic disease virus (EHDV) serotypes are known [22]. Additionally, cell lines derived from *C. nubeculosus* have recently been established [82]. As an initial step to investigate virus inhibitory effects induced by *Wolbachia* infections or transgene-based genetic modifications, the rate of viral proliferation in modified cells can be compared to that reported in previous studies. If inhibition is found, running these assays on lines from both species will aid in understanding the mechanisms behind this inhibition.

### Establishment of laboratory colonies and the logistics of mass release

For SIT, IIT or GM suppression methods to be effective, the target biting midge species must be continuously mass-reared on the scale of tens of thousands of individuals. Whereas replacement strategies do not require inundative releases, they still require the repeated release of substantial numbers of individuals proportional to the natural population. *Culicoides sonorensis* is one of two species of *Culicoides* currently maintained in colonies. Initial colonization of wild-collected *C. sonorensis* can be difficult as well, but there are procedures and protocols in place to aid any attempts [23, 24]. The number of individuals currently being produced (2 million per year) is only constrained by space and labor and can be scaled upward as needed. The colonization of other *Culicoides* species has been attempted, but to date these efforts have been met with limited success or the discontinuation of colonies [22, 83]. The inability to successfully colonize and maintain a species in the laboratory would render these control methods ineffective for that species, making this an urgent area of research need.

For example, the subgenus *Avaritia* contains several primary vector species of pathogens associated with livestock disease on four continents [2]. Two of the most significant vectors in Europe are *Culicoides imicola* and *C. obsoletus*, which transmit BT and Schmallenberg viruses [3]. *Culicoides imicola* is also a significant vector in Africa where it transmits AHSV. Members of this subgenus feed primarily on a variety large mammals and have the ability to breed in dung, thereby tightly linking their life history with susceptible hosts [84]. Attempts to produce viable offspring from field-collected *C. imicola* and *C. obsoletus* have resulted in high oviposition numbers and hatch rates, but high larval mortality [85–87]. Optimization of larval rearing conditions could increase the overall adult yields. Interestingly, results from these studies show an apparent male sex bias from laboratory-reared individuals. The mechanisms behind this bias are unknown, but it may present an additional hurdle in the colonization of these species. Potentially, successful control of one *Culicoides* species using next-generation management tools will signify that substantial resources should be invested in developing and maintaining colonies of these, or other currently intractable midge species of substantial veterinary importance.

### Sex separation technologies

Sex separation can be a bottleneck in the workflow of mass-rearing insects for inundative release, and for certain approaches, it is vital for continued efficacy [30]. In autocidal and genetically-based suppression methods, the unintended release of females will not affect the overall efficacy of the control strategy. With IIT, however, release of *Wolbachia*-infected females could spread the infection into the natural population, nullifying any CI from that strain. There are clear sex differences in *C. sonorensis* that are apparent in the adult and pupal stage [88, 89], although separating the sexes manually is labor-intensive. Sex separation in mosquitoes can be done manually, mechanically, genetically, with insecticide-laden blood meals, or with machine vision technologies [90–93]. Many of these techniques could be modified for use in *Culicoides* midges and evaluated for efficacy and accuracy. Additionally, removal of the females at the adult or pupal stage wastes resources that could be better spent increasing male production. Currently, genetic engineering is the only method that could be used to remove female mosquitoes as larvae. This can be done using a Y-linked fluorescent or visible marker [94, 95], or with conditional sex-lethal genes [62, 96–98]. The remaining males can then be conventionally sterilized, released carrying a transgene or released infected with *Wolbachia*.

### Examination of life-history traits and fitness

Life-history traits also need to be considered and investigated for autocidal, genetically- and *Wolbachia*-based approaches. For example, the number of times females will mate can impact the efficacy of certain methods. Infertility caused by mating with sterile males can be undone by a single mating with a wild-type male. Additionally, traits such as longevity, survivorship, fertility and fecundity can be affected by laboratory-reared insects [93, 99, 100]. The release of males that are less fit and less competitive will subsequently reduce the success of these approaches. Female fitness influences the rate at which a *Wolbachia* strain or transgene will spread in natural populations as part of a replacement approach. Future work needs to include investigating the fitness of individuals from all of the aforementioned strategies in laboratory and field conditions.

### Vector–host–pathogen interactions

The majority of the economically important *Culicoides*-transmitted viruses can be transmitted by multiple species, and there are likely more vector species yet to be identified [1]. Disease-causing pathogens have been isolated from a number of *Culicoides* spp., although dissemination and oral transmission need to be demonstrated in many of these cases [2]. Disentangling incidental infections from the species most important for maintaining transmission should be a priority. Doing so will help assess the practicality of using next-generation control techniques against biting midges and identify where they are most likely to succeed. As multiple vector species can occur sympatrically, the management of a single species may not be sufficient to eliminate or possibly even reduce pathogen transmission. The predominant vector species can also change by region; therefore, these species-specific control measures will be limited in their use geographically. However, even regionally managing virus transmission can reduce the risk of incursions into disease-free areas. Finally, in the event of an exotic virus introduction, such as African horse sickness, having additional tools to use in tandem with pesticides and quarantines will be important.

### Regulatory approval

The use of SIT for the control of insects is bound by few regulatory hurdles in the USA and internationally. Currently, there are no international agreements or regulations on the commercial production and release of sterile insects. That being said, the irradiation procedure is becoming more difficult, with reported delays and denial of shipments of cobalt-60, the common material used for small-scale irradiators. Irradiation using isotopes is subject to federal approval in the USA and by the International Atomic Energy Agency. The use of X-ray radiation to sterilize insects for pest management approaches can address some of the programmatic issues of irradiation procedures. Small-scale X-ray irradiators require less shielding and precautionary measures, are easy to use, are portable and only require an electrical power source [101]. *Wolbachia* approaches are currently regulated in the USA by the Environmental Protection Agency (EPA). Only one IIT approach, for the mosquito *Aedes albopictus*, has been approved to date for commercial sales in the USA [47]. Other IIT and *Wolbachia*-based replacement approaches have been approved for use in multiple countries [49, 102, 103]. Large-scale releases of GM mosquitoes have been carried out in Brazil, Panama and the Cayman Islands [104–106], and the U.S. EPA has issued an experimental use permit for releases in Florida and Texas [107]. Regulatory approval for using *Wolbachia-* and genetically-based approaches are at the purview of each country performing the releases and could require additional approval from a local governing entity.

### Semi-field and small-scale field trials

Information on the short-range dispersal of *Culicoides* midges will be useful in determining localized effectiveness of these strategies. Both the males and females of several *Culicoides* species can disperse 1–3 km, both upwind and downwind, in only a few nights [108, 109]. Male swarms will form near the larval habitat or at a swarm-marker [110, 111]; however, in the case with *C. sonorensis*, mating occurs on or near the host [73]. As the males of this latter species will move to the host, one centralized release point is likely enough to cover a large area for population suppression. In regards to long-range dispersal, *Culicoides* midges possess the ability to disperse via jet streams and thus have the potential to establish long distances from a release site [112, 113]. This behavior will need to be considered during risk assessment of population replacement strategies. Small cage and semi-field trials will also need to be performed for the suggested control approaches, and the work done with mosquitoes can be used as a template in studying *Culicoides* midges. These trials will determine the efficacy of each approach and act as proof of concept by providing an understanding of fitness effects, mating competitiveness, survival and rates of sterility and/or replacement.

### Implementation

While many species of *Culicoides* are associated with disease transmission, here we will highlight two systems for which autocidal and next-generation approaches could be applied, namely population suppression of *Culicoides belkini* and population replacement of *C. sonorensis*. Some species of biting midge species, such as *Culicoides furens* in the Caribbean and *C. belkini* in the South Pacific, are not disease vectors but do adversely impact outdoor activities and tourism, resulting in severe economic impact to the island economies [114–116]. *Culicoides belkini* populations are excellent targets for population suppression because the isolated island distribution likely limits migration between islands. The Louis Malarde Institute recently completed a 600-m^2^ rearing facility to raise *Wolbachia*-infected *Aedes polynesiensis* mosquitoes for population suppression on the Society Islands. This same facility can be used to first colonize and then mass rear the native *C. belkini* (personal communication with the Laboratory Director Herve Bossin). Local populations or small islands can be targeted to prove efficacy of the population suppression or elimination, which will have significant local support by the community and tourist industry. *Culicoides belkini* is the only species of biting midge on certain islands, therefore monitoring the population reduction and detecting re-introductions can be coupled with the local *Ae. aegypti* and *Ae. polynesiensis* monitoring program with relative ease.

Eliminating or reducing a *Culicoides* midge population below a theoretical transmission threshold may not be feasible on a continental scale compared to isolated islands. The alternative is a population replacement strategy to reduce a species vectoral capacity. For BTV, EHDV and vesicular stomatitis virus (VSV) in the USA, the best known vector is *C. sonorensis*. The native range of this species is the central and western USA, primarily west of the Mississippi River [10]. Releasing *Wolbachia*-infected or transgenic *C. sonorensis* with reduced vector competence will not eliminate the population but could help reduce overall virus transmission, even in the presence of other competent vector species [117]. While transmission may not be completely abrogated due to the presence of these other species, pre- and post-release serosurveys of sentinel animals can be used to document any reduction in pathogen transmission [118, 119]. This in turn can shed new light on the importance of *C. sonorensis *in driving the transmission of BTV and EHDV and help identify other vectors that might be important in this process. Moreover, the range of *C*. *sonorensis* is most of the USA west of the Mississippi River; therefore, releasing fewer individuals (compared to inundative releases) is desirable due to the probable high effective population size, as has been shown in *C. imicola*, *C. obsoletus* and *C. brevitarsis* [120–122]. Although the ultimate goal is to eliminate viral transmission, the initial goal of next-generation management practices is to prove their effectiveness against biting midges. Again, success or failure with any approach like this can help inform whether similar approaches will be of value for other *Culicoides* spp.

## Engagement and risk assessment

### Stakeholders

While hemorrhagic disease (HD) caused by BTV and EHDV is usually subclinical or asymptomatic in goats and cattle, it can cause severe symptoms and subsequent death in deer and sheep [123, 124]. These diseases cost the USA roughly USD 125 million annually [125], and this amount likely underrepresents the current economic loss as seen in more recent estimates of the global impact of BT [126]. Commercial deer breeding is the most heavily impacted livestock industry in North America, and in Europe, sheep are most the most susceptible animals [127–129]. Thus, in regards to biting midge control efforts, these farmers are likely to be a primary stakeholder for the use of novel control approaches, especially during disease outbreaks. The organization and structure within these industries will prove beneficial to community engagement efforts to promote novel control approaches at annual meetings and though farmer associations. Secondarily, the cattle and dairy industries have a financial interest in reducing the transmission of these viruses. Although these animals are asymptomatic, trade restrictions and reduced production are still associated with HD [126]. Farms and ranches are often on large plots of privately-owned land, away from cities and towns. The release of sterile or modified males on these farms can offer localized management while maintaining a comfortable distance from the general public. Community-wide surveys and collaboration with local governments in these areas will determine if the isolation of these releases increases their overall acceptance.

### Risk assessment

Autocidal, genetically-, and *Wolbachia*-based methods for vector control can be self-limiting or self-sustaining and the risks associated with each should be weighed alongside any potential benefits prior to release [34, 52, 72]. Proper ecological risk assessments of most species of *Culicoides* midges will be challenging, as certain biological traits remain unknown. For example, of the roughly 1350 species worldwide, the immature stages have been described for < 20% of the species [112]. For the species that have been described, there are limited diagnostic characteristics available, and identification can be difficult or inaccurate. Surveying larval habitats is less important for SIT or IIT, but some genetically engineered control strategies employ this step in risk assessment and monitoring.

For control methods that rely on sterility or incompatibility, heterospecific mating will not affect the outcome of the program; however, for methods that rely on a drive mechanism, gene flow between closely related species can have unintended consequences. There are a number of species complexes within the genus that hinder proper identification and, depending on the relatedness of the species within the complex, could lead to unintended consequences upon release of modified individuals [84]. *Culicoides sonorensis* belongs to a complex of three species, which was historically considered to be five subspecies [89]. If *C. sonorensis* is actively hybridizing with closely related species, the risk of introgression increases substantially. Under laboratory conditions, *C. sonorensis* is able to hybridize with *C. occidentalis* and produce viable offspring for six generations [130]. In nature, pre-zygotic isolation barriers can still exist to keep these species from mating; however, this study [130] shows the potential for gene flow due to the lack of post-zygotic isolation barriers. No natural hybrids have been confirmed, although analyses using more sensitive markers should be conducted [131, 132]. As monitoring programs will be in place to ensure the efficacy of a management program, these can also be used to detect unintended outcomes from releasing modified individuals into the environment.

## Conclusions

As novel strains of *Culicoides*-transmitted viruses continue to spread to new areas [133–135], establishing one or multiple next-generation control methods could provide an effective way to reduce disease transmission. Successful control in one species would provide an outline to adapt these techniques for use in other biting midge pathosystems. Conventional SIT can be the most turnkey option for controlling biting midges, although more work is needed to optimize the radiation dose to minimize any fitness effects attributed to irradiation. Evidence for CI induced by *Wolbachia* infections in *Culicoides* is still needed, but both IIT and *Wolbachia*-based replacement approaches appear promising. No genetic modification of any *Culicoides* midge has been reported; therefore, its use in vector control is likely years away, although developing conditional sex-lethal transgenic lines could be useful for integration of an SIT or IIT approach. Any potential *Wolbachia*- or genetically-induced viral inhibitory effects will need to be demonstrated using *in vitro* and *in vivo* systems. While this review focused mainly on North America and *C. sonorensis*, there are a multitude of *Culicoides* systems that can benefit from these next-generation control techniques. Each of these systems will have its own challenges and hurdles to consider before implementation; however, the ability to preemptively apply the knowledge gained from researching *C. sonorensis* will be invaluable to adapting these tools for use against other biting midge species.

## Data Availability

All data generated or analyzed during this study are included in this published article.

## Abbreviations

AHSV: African horse sickness virus
BT: Bluetongue
BTV: Bluetongue virus
CI: Cytoplasmic incompatibility
CRISPR: Clustered regularly interspaced short palindromic repeats
DENV: Dengue virus
EHD: Epizootic hemorrhagic disease
EHDV: Epizootic hemorrhagic disease virus
GM: Genetically modified
GMO: Genetically modified organism
HD: Hemorrhagic disease
IIT: Incompatible insect technique
RIDL: Release of insects carrying a dominant lethal
SIT: Sterile insect technique
